# Characterization of the prognostic and oncologic values of ITGB superfamily members in pancreatic cancer

**DOI:** 10.1111/jcmm.15990

**Published:** 2020-10-18

**Authors:** Hongkai Zhuang, Zixuan Zhou, Zuyi Ma, Zhenchong Li, Chunsheng Liu, Shanzhou Huang, Chuanzhao Zhang, Baohua Hou

**Affiliations:** ^1^ Department of General Surgery Guangdong Provincial People's Hospital Guangdong Academy of Medical Sciences Guangzhou China; ^2^ Shantou University of Medical College Shantou China; ^3^ The Second School of Clinical Medicine, Southern Medical University Guangzhou China

**Keywords:** CD8^+^ T cells, immune infiltration, ITGB superfamily members, pancreatic cancer, prognosis

## Abstract

Integrin β (ITGB) superfamily members have been reported to play important roles in multiple biological functions in various cancers. However, the prognostic and oncologic values of ITGB superfamily members have not been systematically investigated in pancreatic cancer (PC). In this study, the mRNA expression and biological functions of ITGB superfamily members in PC were evaluated by bioinformatic analysis. Our results demonstrated that ITGB1, ITGB4, ITGB5 and ITGB6 overexpressions were significantly associated with advanced AJCC stage and histologic grade, and worse prognosis in PC. A prognostic signature based on ITGB1, ITGB4, ITGB5 and ITGB6 showed a reliable predictive performance. Furthermore, one CpGs (cg20545410) in promoter region of ITGB1, four (cg18709893, cg15700850, cg20667796 and cg18326022) of ITGB4, two (cg10977398 and cg03518058) of ITGB5 and one (cg23008083) of ITGB6 were negatively associated with their corresponding mRNA expression, and positively associated with prognosis in PC. We also identified TFAP2A as the potential transcription factor for ITGB4, SP1 for ITGB1 and ITGB6, and FHL2 for ITGB5 and ITGB6. ITGB1, ITGB4, ITGB5 and ITGB6 overexpressions were all significantly involved in focal adhesion signalling pathway. ITGB1 and ITGB5 overexpressions also associated with up‐regulation of TGF‐β and WNT signalling pathway, whereas ITGB4 and ITGB6 overexpressions associated with up‐regulation of Notch signalling pathway. Besides, ITGB1, ITGB5 and ITGB6 overexpressions significantly correlated with immunosuppression in PC. In summary, our study investigated the multilevel prognostic and biological values of ITGB superfamily members in PC.

## 1 INTRODUCTION

As one of the most aggressive malignancies, pancreatic cancer (PC) is currently the fourth most common cause of cancer mortality, causing about 4.5% of all cancer associated deaths worldwide.^1^ The overall 5‐year survival rate of PC is only about 8%.^2^ Immunotherapy has been a landmark advancement over the last decade for the treatment of various cancers, such as melanoma, non‐small‐cell lung cancer and hepatocellular carcinoma.^3^, ^4^, ^5^, ^6^, ^7^, ^8^ However, these ICIs have limited effectivities for PC as single agents, due to the limited infiltration of CD8^+^ T cells in the tumour microenvironment (TME) of PC.^9^ Thus, the identification of reliable prognostic biomarkers and drug targets involved in the tumorigenesis, chemoresistance and immunosuppression of PC is necessary for developing more useful chemotherapies and immunotherapies for PC patients.

Integrin‐β (ITGB) superfamily is one of the superfamily of integrins, consisting eight different members in human body.^10^ ITGB superfamily members have been reported to interact with the extracellular matrix (ECM) and the cytoskeleton, and play a critical role in the regulation of various cellular processes including proliferation, carcinogenesis and immune response.^11^, ^12^, ^13^, ^14^ ITGB1 overexpression has been reported to associate with worse prognosis and promotes tumour progression in PC.^15^, ^16^ Meng et al reported that ITGB4 promotes pancreatic carcinogenesis and regulates the MEK1‐ERK1/2 signalling pathway.^17^ Up‐regulation of ITGB4 also promotes epithelial‐mesenchymal transition in PC.^18^ However, the potential biological functions of ITGB5 and ITGB6 in PC have not been explored to date. The whole picture of the prognostic and oncologic characteristics of the entire ITGB superfamily remains poorly explored in PC.

In the present study, for the first time, we comprehensively explored the mRNA expression of ITGB superfamily members and their correlations with prognosis, tumour progression, CpG methylation in promoter regions and immune suppression in PC using biological methods.

## 2 MATERIALS AND METHODS

### 2.1 Acquisition of RNA information

First, we obtained the mRNA expression data and corresponding clinical information for PC from the Cancer Genome Atlas (TCGA, https://cancergenome.nih.gov/) in July 2020. The data of CpG methylation of ITGB superfamily members in TCGA PC cohort were downloaded through MethSurv (https://biit.cs.ut.ee/methsurv/). Among the 177 PC cases in TCGA PC dataset, 171 were cases with OS >1 month. We also downloaded mRNA expression and clinical information for PC from the International Cancer Genome Consortium (ICGC). In addition, GSE62452 dataset and GSE28735 dataset were utilized for differential expression analysis for ITGB superfamily members. ICGC PC dataset, GSE62452 dataset and GES79668 dataset were used for external validation. TCGA PC dataset, GSE79668 dataset, GSE62452 dataset and GSE28735 dataset are all freely available as public databases. Therefore, local ethics approval was not necessary.

### 2.2 Differential expression of ITGB superfamily members

Multiple datasets were utilized to determine the mRNA expression of ITGB superfamily members in PC. GSE62452 dataset was used to evaluated the differential expression of ITGB superfamily members between PC samples (n = 69) and adjacent non‐tumour tissues (n = 61). Then, the differential expression of ITGB superfamily members between PC samples (n = 45) and the corresponding adjacent non‐tumour tissues (n = 45) was investigated using GSE28735 dataset. Finally, the differential expression analysis for ITGB superfamily members in PC was also explored through the Gene Expression Profiling Interactive Analysis (GEPIA; http://gepia.cancerpku.cn/index.html), which was based on the TCGA and GTEx projects.

### 2.3 ITGB superfamily members in AJCC stage and histologic grade

To determine whether ITGB superfamily members would promote PC progression, we investigated the expression level of ITGB superfamily members in different AJCC stage and histologic grade. Histologic grade is used to define tumour differentiation based on pathologic examination. A higher histologic grade indicates a poorer histologic differentiation. The ITGB superfamily members expression differences between the two groups were evaluated using the Wilcoxon test, whereas differences among three or more groups were evaluated using the Kruskal‐wallis test.

### 2.4 Prognostic values of ITGB superfamily members in PC

The prognostic values of ITGB superfamily members in PC were analysed in the TCGA PC dataset using univariate Cox analysis. According to the best cut‐off point obtained from the X‐tile software in version 3.6.1,^19^ Kaplan‐Meier survival curve for overall survival (OS) and recurrence‐free survival (RFS) were further conducted for the prognosis‐associated ITGB superfamily members in univariate Cox analysis. In addition, we performed multivariate Cox analysis to develop a prognostic signature based on the prognosis‐associated ITGB superfamily members for OS in PC. Risk score = ∑ the multivariable Cox regression coefficients × the expression of prognosis‐associated ITGB superfamily members. High‐ and low‐risk groups were determined based on the best cut‐off point obtained from the X‐tile software in version 3.6.1, and KM survival curve was performed using R package survminer. The predictive performance of the prognostic signature was assessed by C‐index and ROC curve for OS. And we also evaluated the differences of RFS among high‐ and low‐risk group through KM survival curve. The discrimination of the signature for RFS was also investigated through the C‐index and ROC curve. Furthermore, ICGC PC dataset, GSE62452 dataset and GSE79668 dataset were used to externally validate the predictive power of the signature for OS in PC.

### 2.5 DNA methylation data in promoter regions of prognosis‐associated ITGB superfamily members in PC

In order to figure out whether the hypomethylation of CpGs in promoter regions (eg the 1st exon, 5’UTR, and TSS) could up‐regulate the expression of prognosis‐associated ITGB superfamily members,^20^, ^21^ we conducted correlation analysis between the mRNA expression level of prognosis‐associated ITGB superfamily members and the methylation level of corresponding CpG in promoter regions using TCGA PC dataset. Furthermore, we evaluated the prognostic value of the significantly correlated CpGs in promoter regions using univariate Cox analysis for OS, which were also validated through KM survival curves for OS.

### 2.6 Prediction of transcription factors and miRNAs for prognosis‐associated ITGB superfamily members

As critical regulators in gene regulation, transcription factor (TF) and miRNA function in the transcriptional and post‐transcriptional levels, respectively.^22^, ^23^ Potential TFs and miRNAs of prognosis‐associated ITGB superfamily members were predicted using Network Analysis (http://www.networkanalyst.ca). The prediction of TFs and miRNAs of prognosis‐associated ITGB superfamily members was based on the RegNetwork database which integrated the existed regulations in multiple databases and the potential regulations based on the TFs binding sites.^24^ TFs or miRNAs that potentially regulated more than two of prognosis‐associated ITGB superfamily members were considered as critical TFs or miRNAs, and selected for co‐expression analysis with their corresponding ITGB superfamily members under the threshold of Pearson correlated coefficient> |0.5|, *P* value <.05. In addition, differential expression analysis and KM survival curves for OS were performed for the co‐expressed critical TFs or miRNAs.

### 2.7 Functional enrichment analysis

Gene set enrichment analysis (GSEA) was conducted to explore the differences of potential biological processes between high and low prognosis‐associated ITGB superfamily members expression groups (*P* value <.05 and a false discovery rate (FDR) <.25). The number of permutations was set at 1000.

### 2.8 Association between prognosis‐associated ITGB superfamily members and immune suppression in the TME of PC

With the help of R package GSVA, single‐sample gene set enrichment analysis (ssGSEA) was performed to evaluate the enrichment levels of immune cells or anti‐tumour effect in the TCGA PC cohort and GSE62452 cohort.^25^ The following immune‐related terms were obtained: Treg cells, TAM, CD8^+^ T cells and cytolytic activity.^26^ Then, correlation analysis between prognosis‐associated ITGB superfamily members and the immune infiltration in PC was performed using Pearson correlation coefficients (|Cor|> 0.20 and *P* value <.05). Then, we evaluated the differences of the immune infiltration between high‐ and low‐risk groups in TCGA PC dataset. Recently, Bailey et al defined four molecular subtypes for PC using 96 PC cases in ICGC PC dataset (validated in the extended set of 266 mRNA arrayed cases), which they termed Squamous, Pancreatic Progenitor, Immunogenic and Aberrantly Differentiated Endocrine Exocrine (ADEX).^27^, ^28^ Therefore, using ICGC PC dataset, we investigated the differences of risk scores among the four subtypes by Bailey et al

### 2.9 Statistical analysis

All statistical analyses were performed using GraphPad prism 8.0 software (GraphPad Software, Inc) and R software (http:///www.r‐project.org/). Correlations were calculated using Pearson correlated coefficient. Group differences were analysed by Wilcoxon test or Kruskal‐wallis test. Data are expressed as means ± SD. *P* values <.05 were considered statistically significant.

## 3 RESULTS

### 3.1 ITGB superfamily members are significantly overexpressed in PC

**Figure 1:**
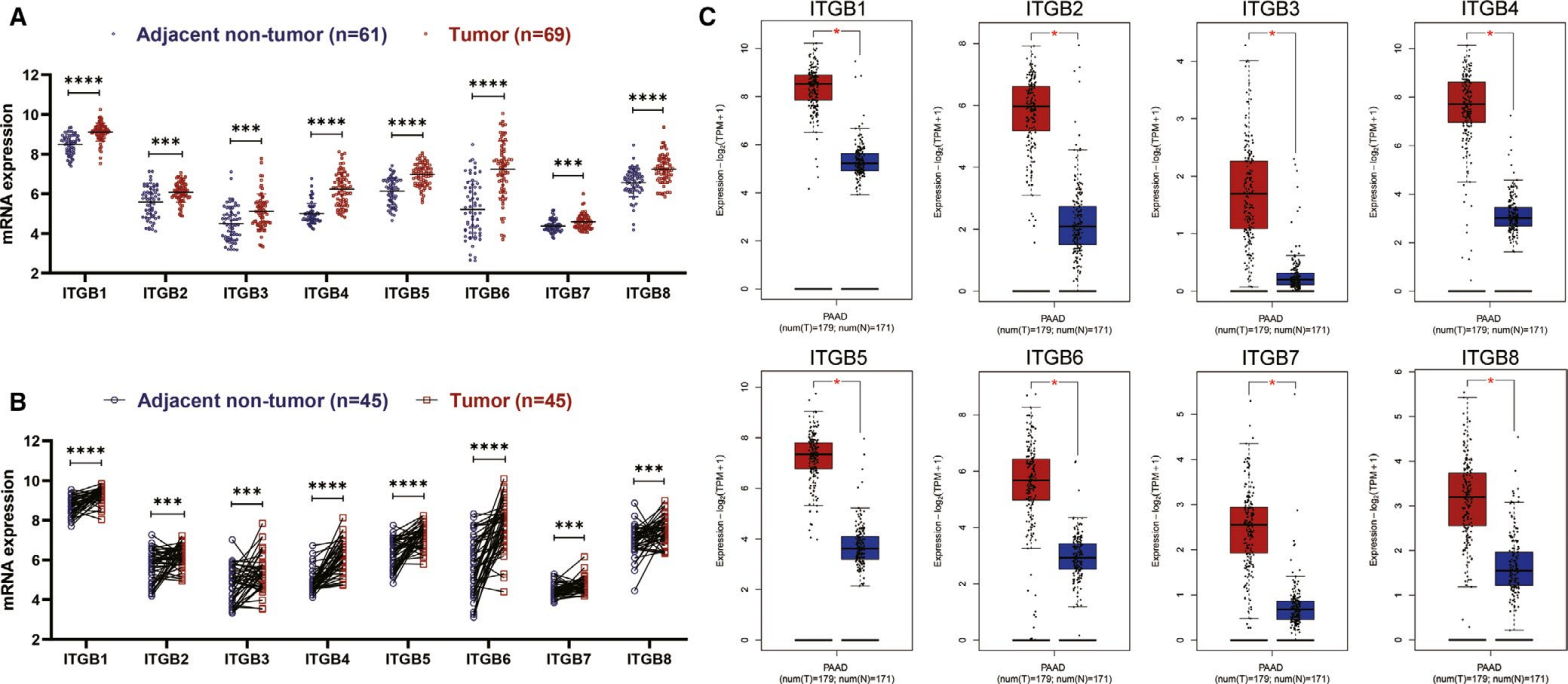
Differential expression analysis of ITGB superfamily members. A, ITGB superfamily members were significantly up‐regulated in PC in GSE62452 dataset. B, ITGB superfamily members were significantly up‐regulated in PC tissues compared with that in the adjacent non‐tumour tissues in GSE28735 dataset. C, ITGB superfamily members were significantly up‐regulated in PC in GEPIA database. (*P value <.05; **P value <.01;***P value <.001; ****P value <.0001)

First, GSE62452 datasets revealed that ITGB superfamily members were significantly up‐regulated in PC tissues compared with adjacent non‐tumour tissues (*P* value <.001) (Figure 1A). Furthermore, based on GSE28735 dataset, ITGB superfamily members were found to be up‐regulated in PC tissues compared with that in the paired adjacent non‐tumour tissues (*P* value <.001) (Figure 1B). In addition, similar results were also shown in the GEPIA database (*P* value <.01), (Figure 1C). Taken together, our study demonstrated that ITGB superfamily members are significantly up‐regulated in PC.

### 3.2 The association between ITGB superfamily members and AJCC stage, histologic grade

**Figure 2:**
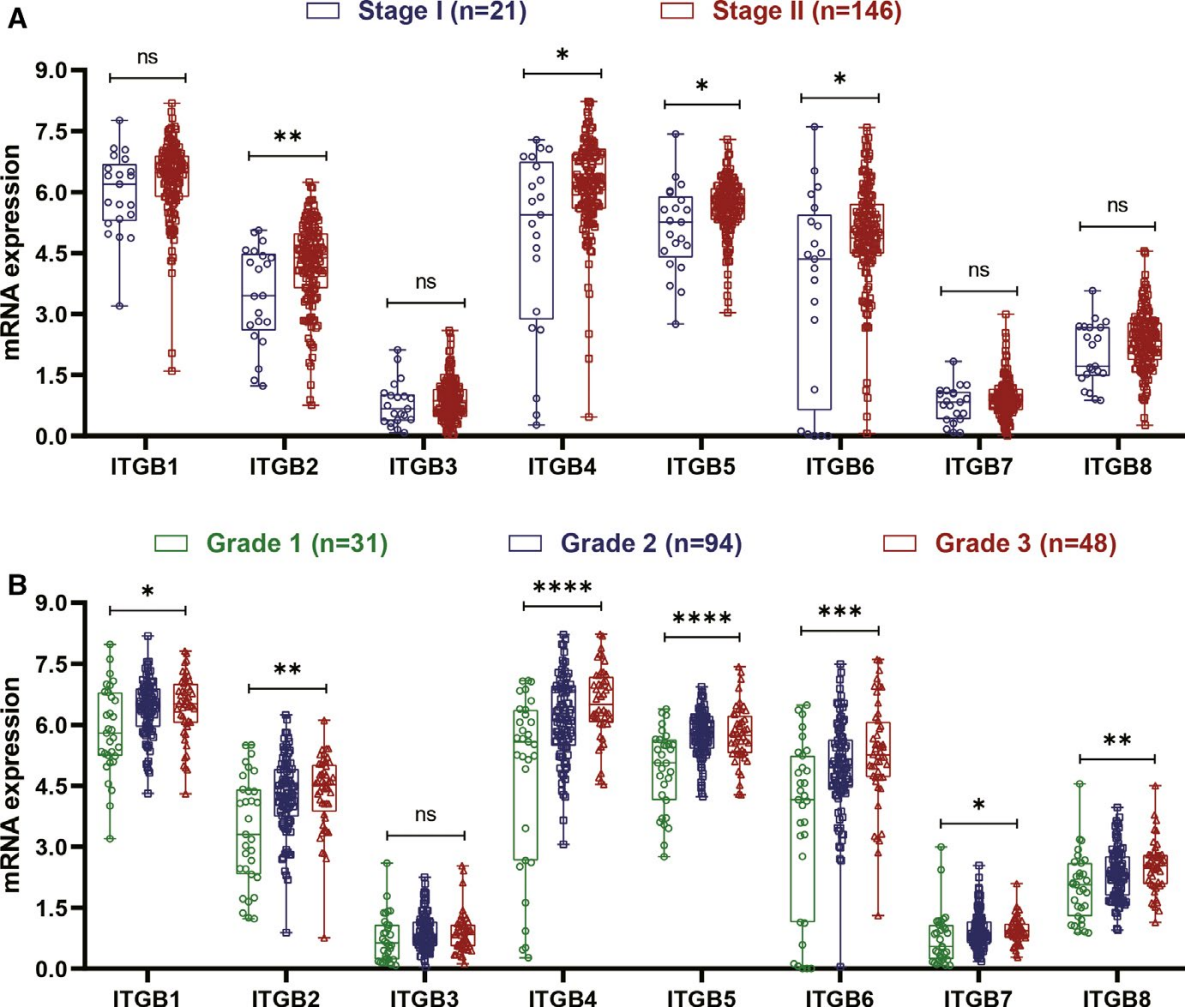
Expression of ITGB superfamily members in AJCC stage and histologic grade. A, The expression of ITGB2, ITGB4, ITGB5 and ITGB6 was significantly increased in patients with AJCC stage II than in patients with AJCC stage II. B, Higher expression of ITGB1, ITGB2, ITGB4, ITGB5, ITGB6, ITGB7 and ITGB8 was also observed in patients with higher histologic grade. (*P value <.05; **P value <.01;***P value <.001; ****P value <.0001)

Patients with AJCC stage II have higher mRNA expression of ITGB2, ITGB4, ITGB5 and ITGB6 compared with those with AJCC stage I. (Figure 2A). Besides, higher mRNA expressions of ITGB 1, ITGB2, ITGB4, ITGB5, ITGB6, ITGB7 and ITGB8 were also observed in patients with higher histologic grade (Figure 2B). These results indicated that ITGB1, ITGB2, ITGB4, ITGB5, ITGB6, ITGB7 and ITGB8 overexpressions were significantly related with both advanced tumour stage and higher grade.

### 3.3 The prognostic value of ITGB superfamily members in PC

**Figure 3:**
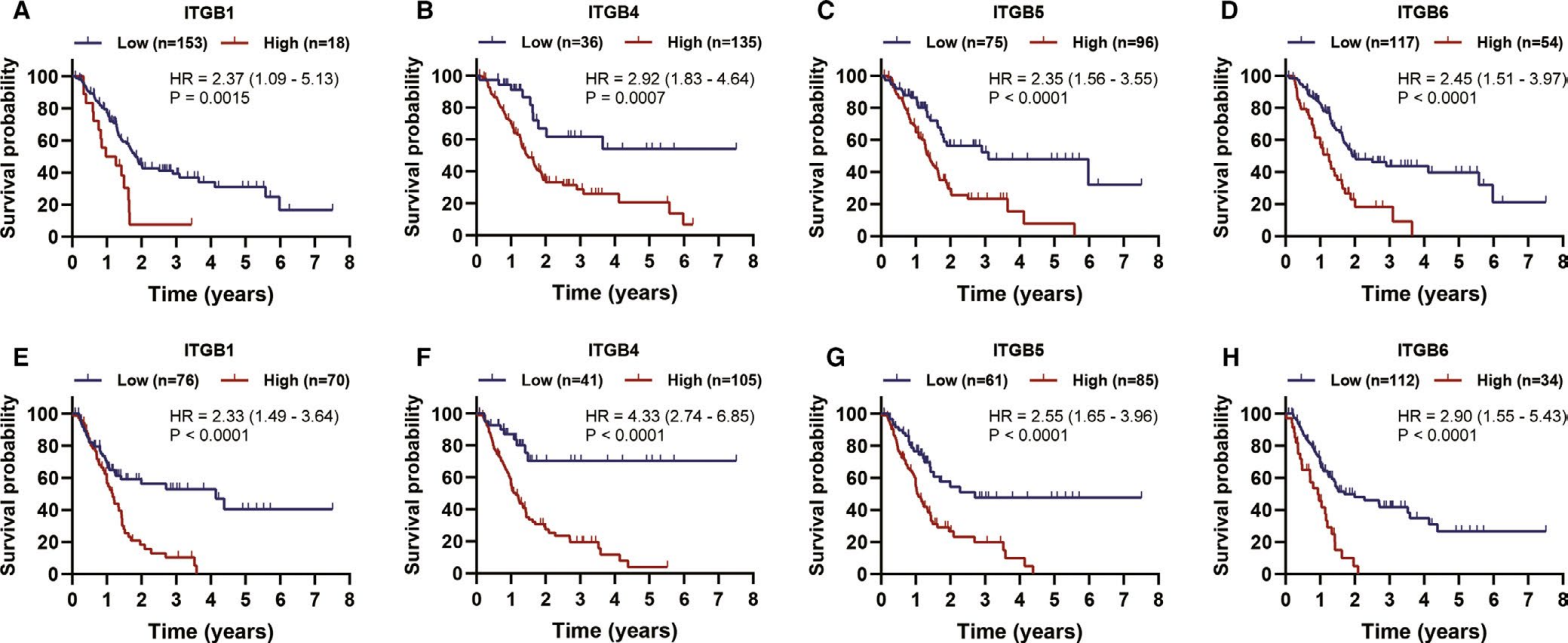
KM survival curves for prognosis‐associated ITGB superfamily members (eg ITGB1, ITGB4, ITGB5 and ITGB6). A‐D, KM survival curves for OS; E‐H, KM survival curves for RFS. (Log‐rank *P* < .05)

**Figure 4:**
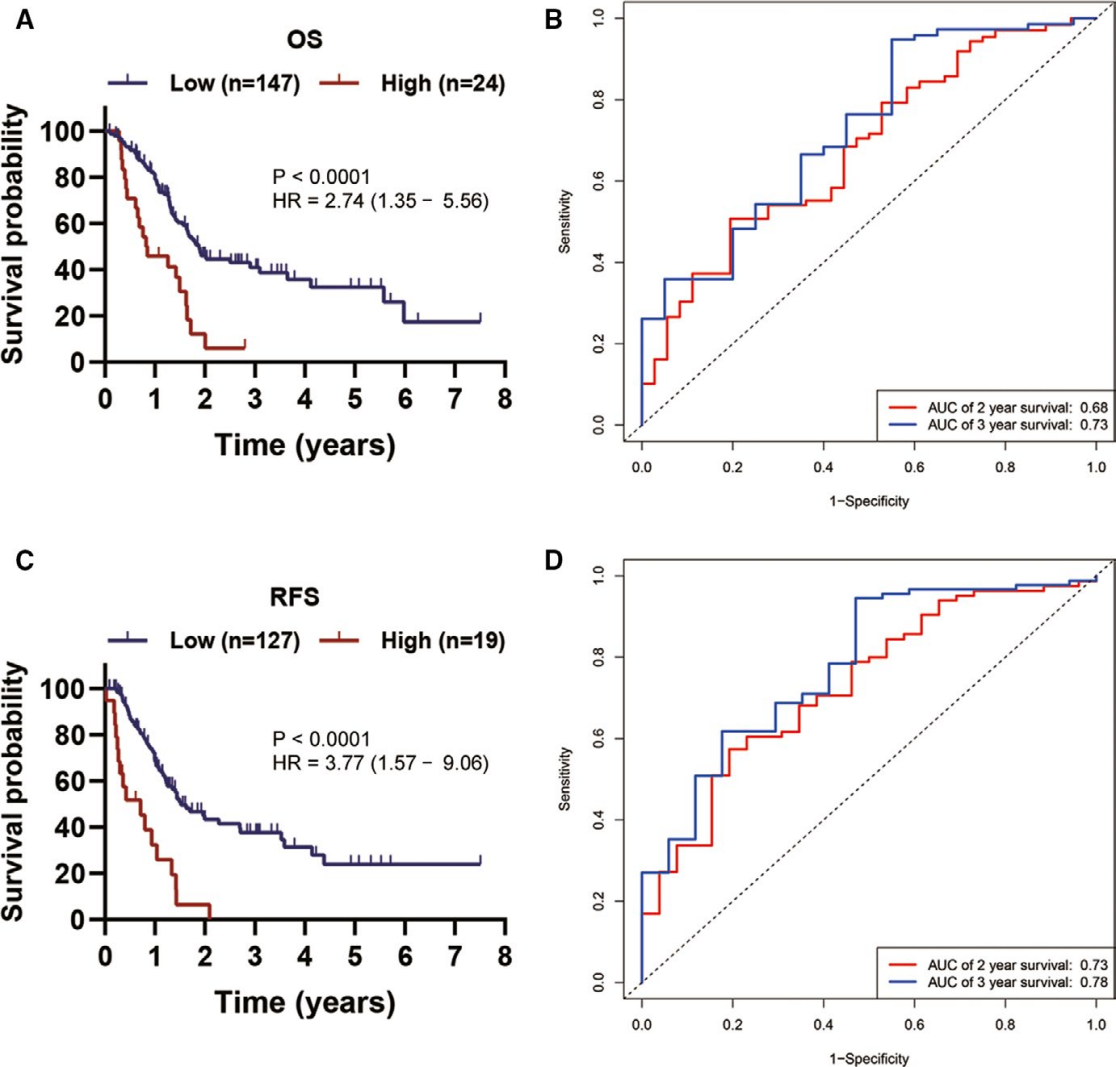
Establishment of a prognostic signature based on ITGB1, ITGB4, ITGB5 and ITGB6. A, KM survival curve showing OS differences between high‐ and low‐risk groups. B, ROC curve analysis of the signature for 2‐ and 3‐year OS prediction of PC patients. C, KM survival curve showing RFS differences between high‐ and low‐risk groups. D, ROC curve analysis of the prognostic signature for 2‐ and 3‐year RFS prediction of PC patients

**Figure 5:**
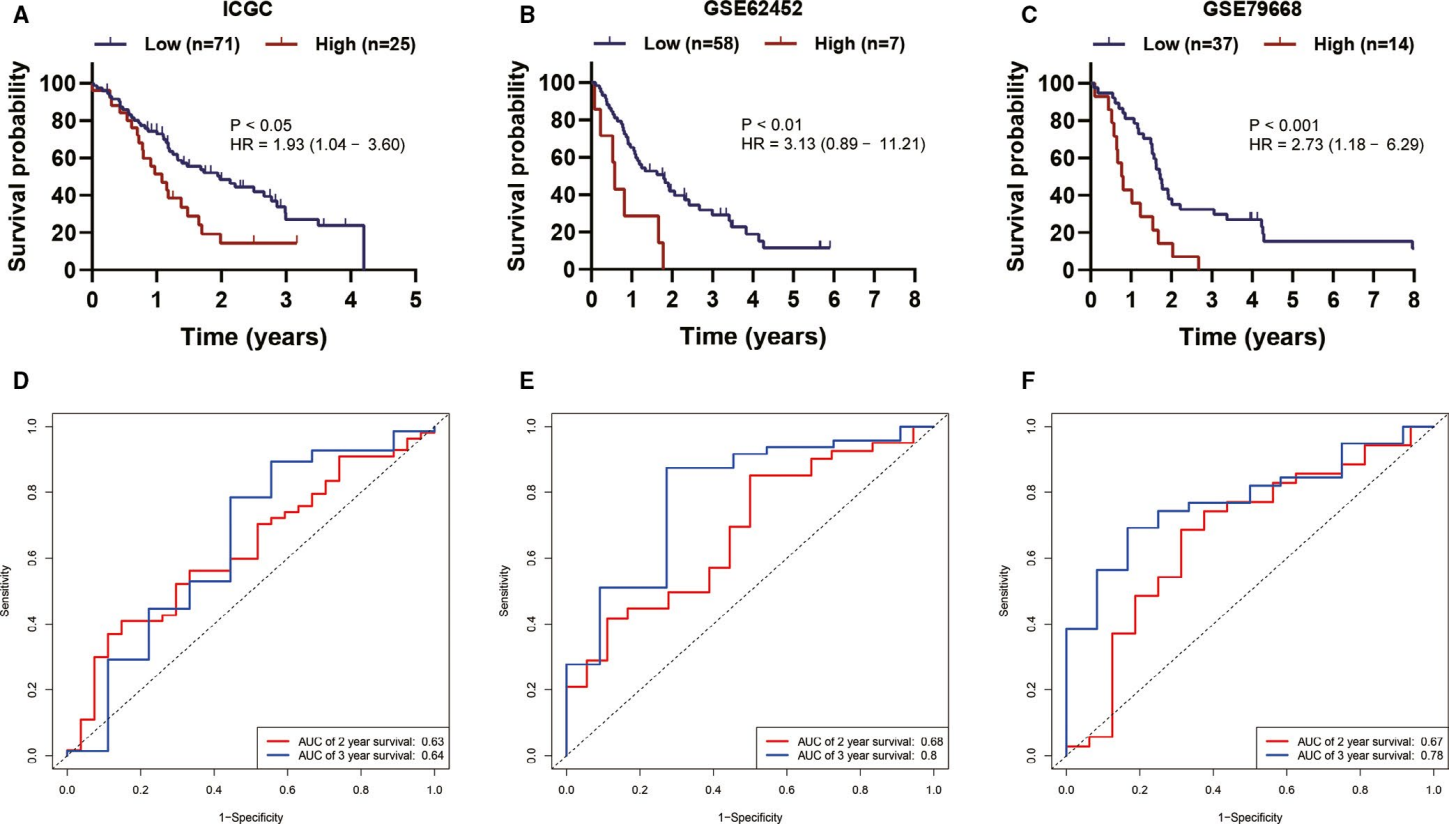
Validation of the prognostic signature based on ITGB1, ITGB4, ITGB5 and ITGB6. A‐C, KM survival curve showing OS differences between high‐ and low‐risk groups. D‐F, ROC curve analysis of the signature for 2‐ and 3‐year OS prediction of PC patients

Using univariate Cox analysis, we demonstrated that increased expression of ITGB1, ITGB4, ITGB5 and ITGB6 was significantly correlated with decreased OS and RFS (Table 1). KM survival curves demonstrated that patients with higher expression of ITGB1, ITGB4, ITGB5 and ITGB6 had a shorter OS or RFS than those with lower expression of ITGB1, ITGB4, ITGB5 and ITGB6 (Figure 3). A prognostic signature was also constructed based on ITGB1, ITGB4, ITGB5 and ITGB6. Risk score = 0.00356 × ITGB1 expression + 0.00125 × ITGB4 expression + 0.00324 × ITGB5 expression + 0.00553 × ITGB6 expression (Table 2). The PC patients in the current study were divided into low‐risk group (a risk score ≦ 1.46) and high‐risk group (a total score >1.46). The KM survival analysis for OS demonstrated that the low‐risk group had longer OS than the high‐risk group (Figure 4A). Of note, the AUC value of the prognostic signature for predicting 2‐ and 3‐year OS was 0.681 and 0.728, respectively (Figure 4B). In addition, KM survival curves for RFS also demonstrated that the low‐risk group had longer RFS than high‐risk group (Figure 4C). And the AUC value of the prognostic signature for predicting RFS increased to 0.780 as follow‐up periods increased (Figure 4D). The C‐indexes for OS and RFS prediction with this prognostic signature were, respectively, 0.644 (95% CI, 0.583 ‐ 0.705) and 0.625 (95% CI, 0.547‐0.703). To further validate these results, the risk score was calculated for patients in ICGC PC dataset, GSE62452 dataset and GSE79668 dataset. And the KM survival curve was conducted for ICGC PC dataset, GSE62452 dataset and GSE79668 dataset, which showed significantly different OS between the high‐ and low‐risk groups (*P* < .05) (Figure 5A‐C). The AUC for OS in ICGC PC dataset was 0.63 at 2 years, and 0.64 at 3 years (Figure 5D). The AUC for OS in GSE62452 dataset was 0.68 at 2 years, and 0.80 at 3 years (Figure 5E). And the AUC for OS in GSE79668 dataset was 0.67 at 2 years, and 0.78 at 3 years (Figure 5F). Taken together, our results indicated a good performance of the prognostic signature for PC prognosis prediction, especially for PDAC.

**Table 1:** Univariate Cox analysis of ITGB superfamily members for PC prognosis

Gene	Overall survival			Recurrence‐free survival		
	HR	95% CI	*P*	HR	95% CI	*P*
ITGB1	1.008	1.003‐1.012	**.00036**	1.009	1.004‐1.013	**.00021**
ITGB2	1.004	0.990‐1.018	.61	1.002	0.987‐1.018	.80
ITGB3	1.275	1.005‐1.618	**.046**	1.230	0.965‐1.566	.094
ITGB4	1.004	1.002‐1.007	**.0058**	1.007	1.004‐1.010	**.000018**
ITGB5	1.013	1.006‐1.020	**.00011**	1.013	1.006‐1.019	**.00027**
ITGB6	1.010	1.006‐1.015	**<.0001**	1.012	1.007‐1.017	**<.0001**
ITGB7	1.044	0.825‐1.321	.72	1.067	0.858‐1.327	.56
ITGB8	1.032	0.983‐1.084	.20	1.039	0.990‐1.090	.12

Bold values indicates *P* values < 0.05 were considered statistically significant.

**Table 2:** Multivariate Cox regression analyses for construction of predictive model signature for OS of PC patients

Gene	Coefficient	HR	HR 95% Low	HR 95% High	*P* value
ITGB1	0.00356	1.004	0.998	1.009	.235
ITGB4	0.00125	1.001	0.998	1.005	.493
ITGB5	0.00324	1.003	0.993	1.013	.53
ITGB6	0.00553	1.006	0.998	1.013	.149

### 3.4 DNA methylation in promoter regions of ITGB1, ITGB4, ITGB5 and ITGB6

**Figure 6:**
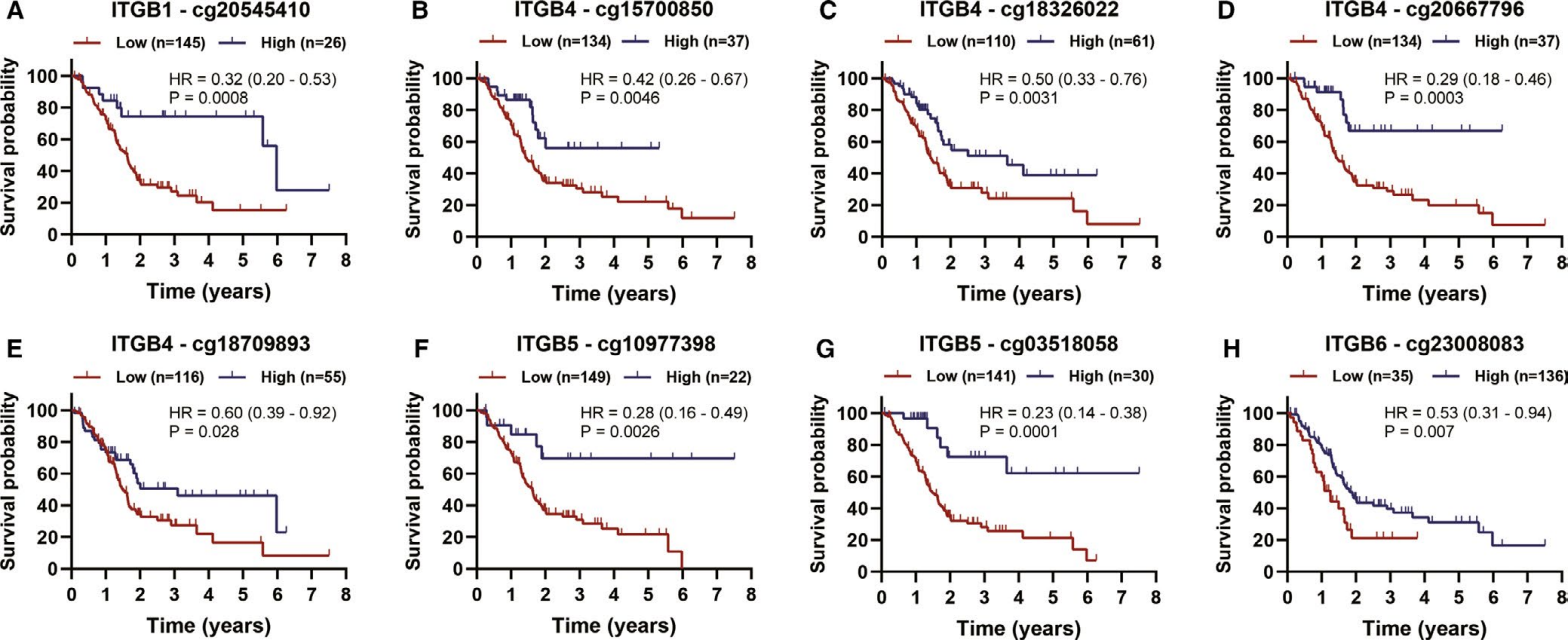
KM survival curves for OS of prognosis‐associated CpG in promoter regions that negatively correlated with their corresponding mRNA expression

First, correlation analysis demonstrated that two CpG in promoter region of ITGB1, six CpG in promoter region of ITGB4, three CpG in promoter region of ITGB5 and one CpG in promoter region of ITGB6 were negatively associated with their corresponding mRNA expression (Table 3). In addition, among these 12 significantly correlated CpG in promoter regions, eight showed significant unfavourable prognosis value for PC based on univariate Cox analysis and KM survival analysis (Table 4; Figure 6).

**Table 3:** Correlation between CpG methylation in promoter regions and mRNA expression of prognosis‐associated ITGB superfamily members

Gene	CpG	Cor	*P*
ITGB1	TSS200;TSS1500‐Island‐cg23837756	−0.21	.0041
ITGB1	5'UTR‐Open_Sea‐cg20545410	−0.25	.0007
ITGB4	TSS1500‐N_Shore‐cg12146151	−0.24	.0015
ITGB4	5'UTR;TSS200‐S_Shelf‐cg18709893	−0.28	.0002
ITGB4	TSS1500;5'UTR‐S_Shore‐cg27346988	−0.43	<.0001
ITGB4	Body;1stExon‐S_Shelf‐cg15700850	−0.44	<.0001
ITGB4	TSS1500;5'UTR‐S_Shelf‐cg20667796	−0.65	<.0001
ITGB4	TSS1500;5'UTR‐S_Shelf‐cg18326022	−0.74	<.0001
ITGB5	TSS1500‐Island‐cg10977398	−0.2	.0075
ITGB5	TSS1500‐Island‐cg15119377	−0.24	.0011
ITGB5	TSS1500‐Island‐cg03518058	−0.4	<.0001
ITGB6	TSS1500‐Open_Sea‐cg23008083	−0.32	<.0001

**Table 4:** Prognosis‐associated CpGs in promoter regions of ITGB superfamily members based on univariate Cox analysis

Gene	CpG	HR	95% CI	*P*
ITGB1	TSS200;TSS1500‐Island‐cg23837756	2.96E‐07	5.98E‐31‐1.46E + 17	.589
ITGB1	5'UTR‐Open_Sea‐cg20545410	0.199	0.045‐0.88	**.034**
ITGB4	TSS1500‐N_Shore‐cg12146151	0.356	0.02‐6.34	.482
ITGB4	5'UTR;TSS200‐S_Shelf‐cg18709893	0.065	0.0053‐0.811	**.034**
ITGB4	TSS1500;5'UTR‐S_Shore‐cg27346988	0.609	0.096‐3.876	.599
ITGB4	Body;1stExon‐S_Shelf‐cg15700850	0.08	0.009‐0.696	**.022**
ITGB4	TSS1500;5'UTR‐S_Shelf‐cg20667796	0.107	0.017‐0.689	**.019**
ITGB4	TSS1500;5'UTR‐S_Shelf‐cg18326022	0.182	0.0451‐0.734	**.017**
ITGB5	TSS1500‐Island‐cg10977398	1.46E‐09	3.73E‐15‐0.00057	**.002**
ITGB5	TSS1500‐Island‐cg15119377	1.97E‐17	2.58E‐36‐150.01	.083
ITGB5	TSS1500‐Island‐cg03518058	3.49E‐07	1.38e‐11‐0.009	**.004**
ITGB6	TSS1500‐Open_Sea‐cg23008083	0.075	0.009‐0.635	**.017**

Bold values indicates *P* values < 0.05 were considered statistically significant.

### 3.5 Prediction of transcription factors for IGTB1, ITGB4, ITGB5 and ITGB6

**Figure 7:**
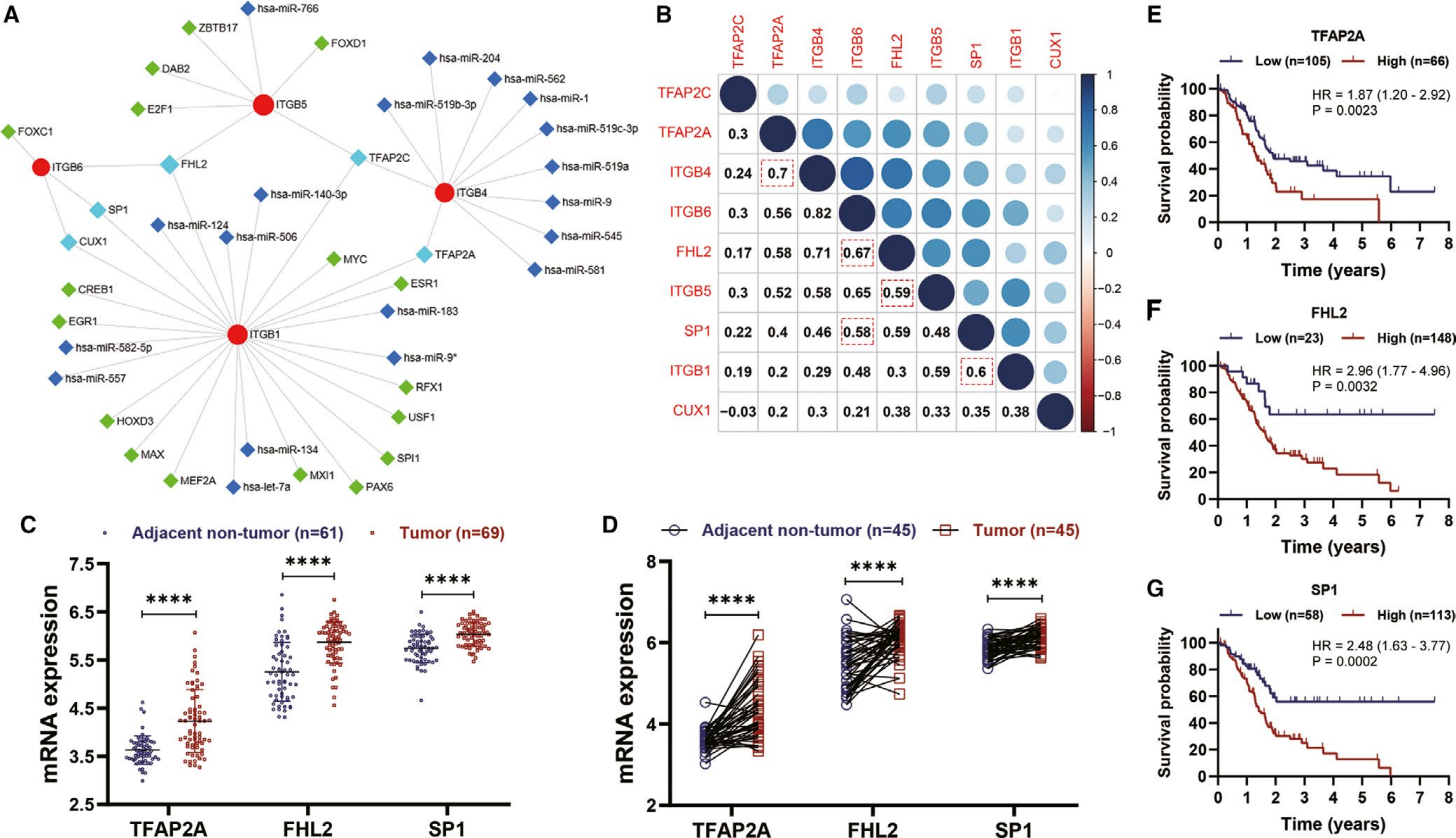
Prediction of transcription factors for ITGB1, ITGB4, ITGB5 and ITGB6. A, The predicted network of TF and miRNA for ITGB1, ITGB4, ITGB5 and ITGB6. B, Correlation analysis among ITGB1, ITGB4, ITGB5 and ITGB6, and their corresponding predicted TFs. C‐D. TFAP2A, FHL2 and SP1 were significantly up‐regulated in PC. E‐G, TFAP2A, FHL2 and SP1 overexpressions were significantly associated with worse OS in PC

Based on the RegNetwork database, no miRNA was predicted to synchronously correlated with ITGB1, ITGB4, ITGB5 and ITGB6, or as least two of them (Figure 7A). Of note, ITGB1 expression was predicted to be potentially regulated by TFAP2A, TFAP2C, FHL2, SP1 and CUX1 (Figure 7A). ITGB4 expression might be potentially regulated by TFAP2A and TFAP2C (Figure 7A). ITGB5 expression might be potentially regulated by TFA2PC and FHL2. And SP1 and FHL2 were predicted to regulate ITGB6 (Figure 7A). Among these five TFs, TFAP2A expression was positively correlated with ITGB4 expression, SP1 expression was positively correlated with ITGB1 and ITGB6 expression, and FHL2 expression was positively correlated with ITGB5 and ITGB6 expression (Cor > 0.50, *P* < .05) (Figure 7B). Furthermore, among these five TFs, TFAP2A, FHL2 and SP1 expressions were significantly up‐regulated in PC tissues (Figure 7C‐D). In addition, KM survival analysis demonstrated that overexpression of TFAP2A, FHL2 and SP1 was significantly associated with worse OS in PC (Figure 7E‐G). Taken together, our study implied that TFAP2A might promote the transcription of ITGB4, SP1 might synchronously promote ITGB1 and ITGB6 expressions, and FHL2 might synchronously promote ITGB5 and ITGB6 expressions.

### 3.6 Functional enrichment analysis for ITGB1, ITGB4, ITGB5 and ITGB6

**Figure 8:**
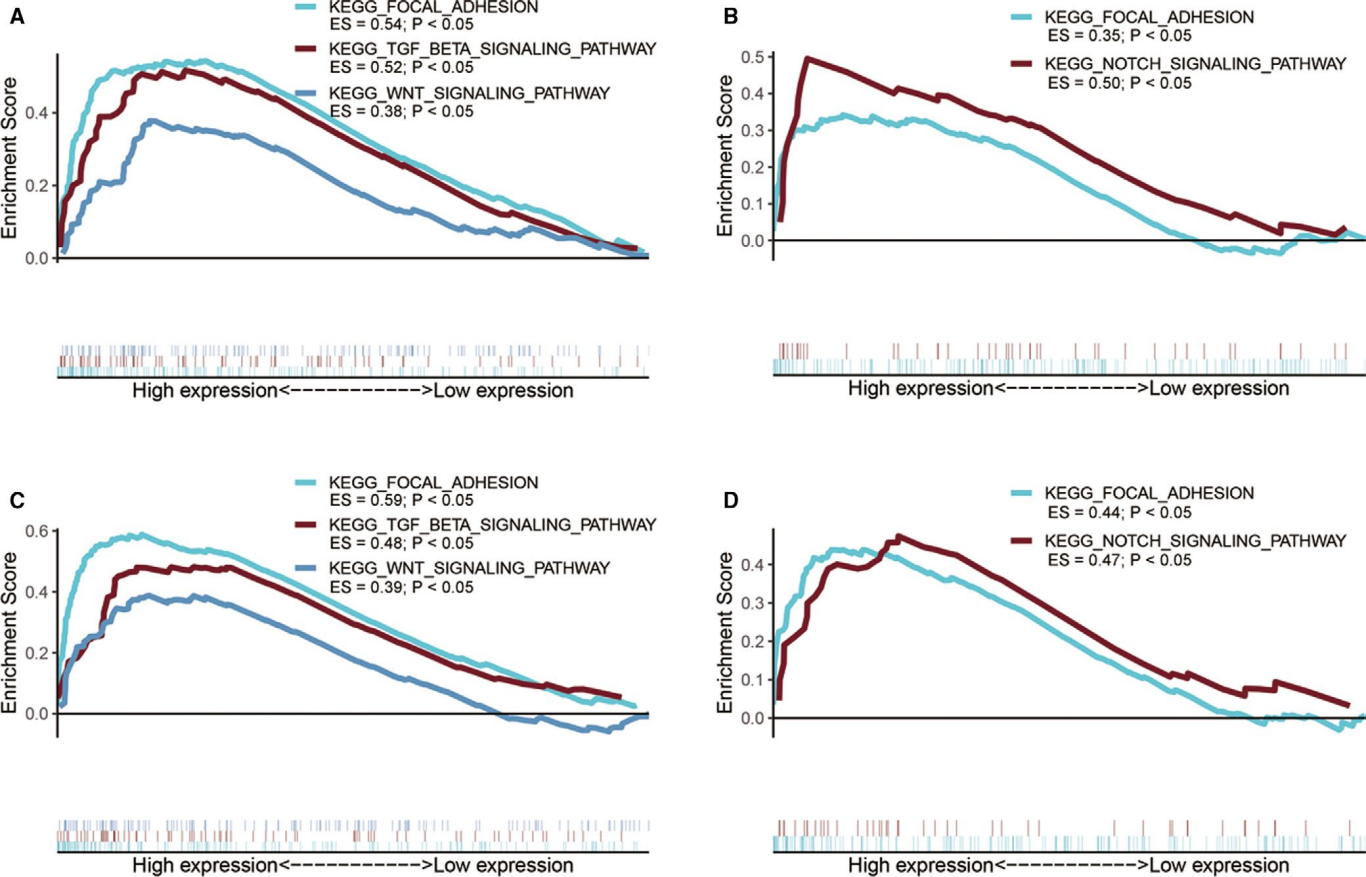
Functional enrichment analysis for ITGB1, ITGB4, ITGB5 and ITGB6. A, GSEA results of ITGB1 expression in PC patients. B, GSEA results of ITGB4 expression in PC patients. C, GSEA results of ITGB5 expression in PC patients. D, GSEA results of ITGB6 expression in PC patients

Overexpression of ITGB1, ITGB4, ITGB5 and ITGB6 was all significantly associated with up‐regulation of focal adhesion‐related gene sets (Figure 8). Besides, both ITGB1 and ITGB5 overexpressions were involved in TGF‐β and WNT signalling pathways (Figure 8A,C). Both ITGB4 and ITGB6 overexpressions were involved in Notch signalling pathway (Figure 8B,D). These results implied that ITGB1, ITGB4, ITGB5 and ITGB6 overexpressions provided necessary support for tumorigenesis and invasion in PC.

### 3.7 ITGB1, ITGB5 and ITGB6 overexpressions correlated with immune suppression in PC

**Figure 9:**
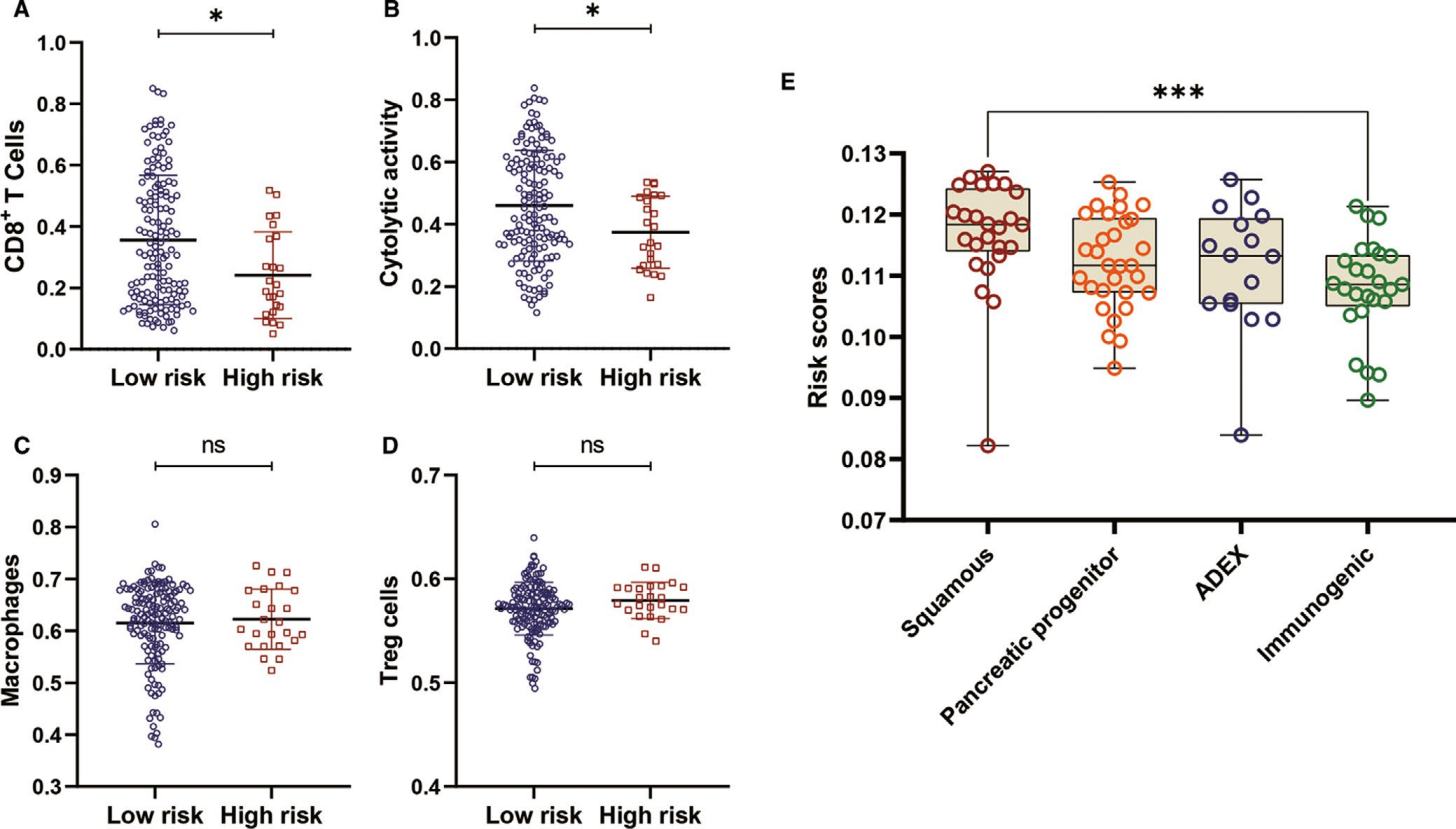
Differences of immune‐related terms enrichment between high‐ and low‐risk groups. Differences of risk scores among four subtypes by Bailey et al A. Patients in low‐risk group had significant higher infiltration CD8^+^ T cells compared with those in high‐risk group. B. Patients in low‐risk group had significant higher cytolytic activity of CD8^+^ T cells compared with those in high‐risk group. C‐D, There is no differences of macrophages and Treg cells infiltrations between high‐ and low‐risk groups. E. Patients in patients in Immunogenic subtype had significant lower‐risk scores than those in squamous subtypes

ssGSEA demonstrated that overexpression of ITGB6 significantly correlated with less infiltration and cytolytic activity of CD8^+^ T cells, inducing immunosuppression in PC (Table 5). And overexpression of ITGB1 and ITGB5 significantly associated with higher infiltration of TAM in PC (Table 5). Besides, ITGB1 overexpression significantly associated with higher infiltration of Treg cells in PC (Table 5). Both Treg cells and TAM also induced immune suppression in the tumour microenvironment of PC.^29^ In addition, our study also identified less infiltration and cytolytic activity of CD8^+^ T cell in PC tissues of patients in high‐risk group (*P* value <.05) (Figure 9A‐B). By analysing ICGC PC dataset, we investigated the differences of the risk scores among the four subtypes defined by Bailey et al, which were squamous, pancreatic progenitor, immunogenic and aberrantly differentiated endocrine exocrine (ADEX). We found patients in immunogenic subtype had significant lower‐risk scores than those in squamous subtypes (Figure 9E). These results suggest that overexpression of ITGB1, ITGB5, and ITGB6 and high‐risk score may be associated with immunosuppression in PC. Taken together, our predictive signature may facilitate clinicians to identify patients with less immunosuppression.

**Table 5:** Correlation between prognosis‐associated ITGB superfamily members and immune‐related terms based on ssGSEA

Dataset	Gene	CD8^+^ T cells		Cytolytic activity		TAM		Treg cells	
		Cor	*P*	Cor	*P*	Cor	*P*	Cor	*P*
TCGA	ITGB1	0.0056	.46	0.019	.81	0.37	**<.0001**	0.51	**<.0001**
	ITGB4	−0.39	**<.0001**	−0.33	**<.0001**	−0.066	.38	−0.083	.27
	ITGB5	−0.16	.03	−0.093	.22	0.35	**<.0001**	0.29	**.0001**
	ITGB6	−0.22	**.0039**	−0.22	**.004**	0.093	.22	0.15	.051
GSE62452	ITGB1	−0.12	.31	−0.023	.85	0.32	**.0077**	0.47	**<.0001**
	ITGB4	−0.22	.067	−0.17	.17	0.17	.16	0.22	.076
	ITGB5	−0.087	.48	−0.093	.45	0.43	**.0003**	0.24	.051
	ITGB6	−0.33	**.0062**	−0.28	**.019**	0.17	.15	0.19	.11

Bold values indicates *P* values < 0.05 were considered statistically significant.

## 4 DISCUSSION

The present study indicated that ITGB1, ITGB4, ITGB5 and ITGB6 expressions significantly associated with higher AJCC stage, advanced histologic grade, worse OS and RFS. Besides, we also established a prognostic signature based on ITGB1, ITGB4, ITGB5 and ITGB6, which showed optimal performance with high C‐indexes [0.644 (95% CI, 0.583‐0.705) for OS and 0.625 (95% CI, 0.547‐0.703) for RFS]. Patients with lower‐risk score had significantly superior OS and RFS than those with higher risk score. The AUC of the ROC curve analysis of this signature for OS and RFS was both up to 0.70 as follow‐up periods increased, indicating its satisfactory predictive power for PC. Besides, patients in low‐risk group had significant higher infiltration and cytolytic activity of CD8^+^ T cells compared with those in high‐risk group. Lower‐risk scores significantly associated with immunogenic subtype by Bailey et al^27^ These results suggest that ITGB1, ITGB4, ITGB5 and ITGB6 may serve as potential prognostic biomarkers for PC. Moreover, the prognostic signature based on ITGB1, ITGB4, ITGB5 and ITGB6 may facilitate clinicians to identify more aggressive and immunosuppressive tumours and make more individually appropriate therapeutic decisions.

Alterations of DNA methylation were reported to be the first detectable neoplastic changes correlated with carcinogenesis.^30^ In addition, hypomethylation of gene promoters has been well described, which permits transcriptional activation of proto‐oncogenes, retrotransposons and malignant encoding proteins involved in malignant cell metastasis.^31^, ^32^, ^33^ Li et al reported that ITGB4 might be up‐regulated by its promoter hypomethylation in colon cancer.^34^ Wang et al demonstrated that ITGB6 was obviously up‐regulated and hypomethylated in the carcinogenesis of intrahepatic cholangiocarcinoma.^35^ However, up to now, studies about the DNA methylation in promoter regions of ITGB1, ITGB4, ITGB5 and ITGB6 in pancreatic carcinogenesis have not been performed to date. For the first time, our study evaluated the associated between the methylation level of CpGs in promoter region and the corresponding mRNA expression. Hypomethylation of cg20545410 was significantly associated with ITGB1 overexpression and worse OS in PC. Hypomethylation of cg18709893, cg15700850, cg20667796 and cg18326022 was significantly associated with ITGB4 overexpression and worse OS in PC. Hypomethylation of cg10977398 and cg03518058 was significantly associated with ITGB5 overexpression and worse OS in PC. Hypomethylation of cg23008083 was significantly associated with ITGB6 overexpression. These results, therefore, support the hypothesis of CpGs hypomethylation in promoter region being potential positive regulators of ITGB1, ITGB4, ITGB5 and ITGB6 expressions in PC, which remains to be proved experimentally in the future studies.

Based on the RegNetwork database, our study identified SP1 as the potential TF for ITGB1 and ITGB6, FHL2 as the potential TF for ITGB5 and ITGB6, and TFAP2A as the potential TF for ITGB4. All of these three TFs (SP1, FHL2 and TFAP2A) were obviously up‐regulated and associated with worse OS in PC. A previous study by Lu et al reported that SNRK complexed with SP1 bound to an SP1‐binding motif in the ITGB1 promoter, resulting in enhanced ITGB1 expression in endothelial cells.^36^ In this study, SP1 overexpression was significantly correlated with ITGB1 overexpression and worse OS in PC. Similarly, we supposed that SP1 might enhance ITGB1 expression in PC. The current study also proposed that TFAP2A might enhance ITGB4 expression, FHL2 might enhance ITGB5 expression, and SP1 and FHL2 might enhance ITGB6 expression in PC. However, up to now, no experimental studies have been conducted to explore these potential correlations in PC or any other kind of cancers. Thus, our study provided provides deeper insight into the potential mechanisms of ITGB1, ITGB4, ITGB5 and ITGB6 in pancreatic carcinogenesis.

Through GSEA, we figured out that ITGB1, ITGB4, ITGB5 and ITGB6 were all significantly involved in focal adhesion signalling pathway. The formation and turnover of focal adhesion are essential for cell migration and invasion.^37^ Previous studies revealed that focal adhesion signalling pathway plays a crucial role in the process of epithelial to mesenchymal transition (EMT) in PC.^38^ Yang et al demonstrated that loss of ITGB1 in breast cancer cells decreased the level of phosphorylated FAK.^39^ Inhibition of ITGB1 also attenuates the tumorigenesis of ovarian cancer cells and contributes to bevacizumab anticancer therapy through focal adhesion signalling pathway.^40^ Chen et al reported that ITGB4 mediates the activation of focal adhesion signalling pathway in ovarian cancer.^41^ ITGB4 was also reported to induce tumorigenesis of colon cancer though activating focal adhesion signalling pathway.^42^ Li et al reported that ITGB4 triggers FAK to promote migration, invasion and EMT process in hepatocellular carcinoma.^43^ Wang et al demonstrated that ITGB5 activates focal adhesion signalling pathway in breast and cervical cancer cell glycolysis alteration and induces cisplatin resistance.^44^ Similarly, our study, for the first time, implied that ITGB1, ITGB4, ITGB5 and ITGB6 overexpressions were significantly associated with up‐regulation of focal adhesion signalling pathway, suggesting the potential role of ITGB1, ITGB4, ITGB5 and ITGB6 in focal adhesion signalling pathway in PC. Experimental study will performed to explore the role of ITGB1, ITGB4, ITGB5 and ITGB6 in focal adhesion signalling pathway during pancreatic carcinogenesis in future works.

In addition, ITGB1 and ITGB5 expressions were also positively associated with TGB‐β signalling pathway and WNT signalling pathway. Studies by Yang et al have also shown that ITGB1 overexpression in breast cancer led to activation of WNT signalling pathway.^39^ ITGB1 down‐regulation was also reported to diminish the role of TGF‐β action on ovarian cancer cells.^45^ Both TGF‐β and WNT signalling were previously reported to be implicated in pancreatic carcinogenesis by promoting the EMT process.^46^, ^47^ Ren et al reported that TGF‐β could promote the migration of PC cells by inducing EMT during the activation of WNT signalling pathway.^48^ The activation of TGF‐β signalling pathway promotes Treg cells recruitment and TAMs polarization and induces immunosuppression in the TME.^49^, ^50^ Additionally, existing studies suggests a critical positive feedback loop between the activation of EMT and the infiltration of TAMs in cancers, which, in turn, induce immune evasion and suppression in the TME.^51^, ^52^, ^53^ Besides, the activation of EMT has been reported to promote resistance to immunotherapies.^54^, ^55^ Interestingly, overexpression of ITGB1 and ITGB5 was significantly associated with higher infiltration of TAMs in PC. Taken together, our study proposed that ITGB1 and ITGB5 might promote the infiltration of TAMs in PC through TGF‐β/WNT‐mediated EMT, which may, in turn, induce the immune suppression in PC. Consequently, we proposed that targeting ITGB1 or ITGB5 may block EMT, which could be a promising therapeutic strategy complementary to current immunotherapies and efficiently improve their effectiveness.

In this study, overexpression of ITGB6 was significantly associated with up‐regulation of Notch signalling pathway. Besides, ITGB6 overexpression was significantly associated with lower infiltration and cytolytic activity of CD8^+^ T cells in PC. Increasing evidences revealed that the excessive action of Notch signalling pathway affects cancer development, and specifically PC progression. Activation of Notch signalling pathway also promotes EMT, which is associated with immune checkpoint inhibitor resistance.^56^ Increasing evidences suggested the critical role of Notch signalling pathway in the suppression of CD8^+^ T cells response in various cancers.^57^ These results suggested that ITGB6 overexpression may suppress the infiltration and anti‐tumour effect of CD8^+^ T cells in PC through Notch signalling pathway, suggesting ITGB6 as a therapeutic target of Notch signalling pathway.

To the best of our knowledge, we are the first to comprehensively describe the prognostic and oncologic values of the mRNA expression and the DNA methylation in promoter regions of ITGB superfamily members in PC. Moreover, for the first time, we demonstrated that ITGB1, ITGB5 and ITGB6 overexpressions may promote immune suppression in PC. However, some limitations should be acknowledged in this study. The first limitation of this study is the lack of experimental validation and externally clinical cohort validation. Second, the study outcomes were largely dependent on the quality of data from publicly available databases.

## 5 CONCLUSION

In the current study, we described the prognostic value and potential biological functions of ITGB superfamily members in PC, providing insights for further investigation of ITGB superfamily members as potential targets in PC.

## CONFLICT OF INTEREST

All authors declare that they have no conflicts of interest.

## AUTHOR CONTRIBUTIONS

**Hongkai Zhuang:** Conceptualization (equal); Data curation (lead); Formal analysis (lead); Investigation (lead); Methodology (lead); Project administration (lead); Resources (lead); Software (lead); Supervision (lead); Validation (lead); Visualization (lead); Writing‐original draft (lead); Writing‐review & editing (lead). **Zixuan Zhou:** Writing‐original draft (supporting); Writing‐review & editing (supporting). **Zuyi Ma:** Writing‐original draft (supporting); Writing‐review & editing (supporting). **Zhenchong Li:** Writing‐original draft (supporting); Writing‐review & editing (supporting). **Chunsheng Liu:** Writing‐original draft (supporting); Writing‐review & editing (supporting). **Shanzhou Huang:** Writing‐original draft (supporting); Writing‐review & editing (supporting). **Chuanzhao Zhang:** Conceptualization (equal); Funding acquisition (equal); Project administration (equal); Resources (equal). **Baohua Hou:** Conceptualization (equal); Funding acquisition (equal); Project administration (equal); Resources (equal).

## Data Availability

The authors confirm that the data supporting the findings of this study are available within the article and its supplementary.
